# Identification and phylogenetic analysis of contagious ecthyma virus from camels (*Camelus dromedarius*) in Iran

**DOI:** 10.4102/ojvr.v84i1.1257

**Published:** 2017-03-24

**Authors:** Ahmad Oryan, Mahboobe Mosadeghhesari, Saeed Zibaee, Ali Mohammadi

**Affiliations:** 1 Department of Pathobiology, Shiraz University, Iran; 2 Razi Vaccine and Serum Research Institute, Mashhad, Iran

## Abstract

Contagious ecthyma is a highly contagious disease affecting domestic and wild ruminants such as sheep, goats and camels. The identification and characterisation of a parapoxvirus (PPV) infecting camels is described here. The virus was detected in dromedary camels (*Camelus dromedarius*) from Kerman and Shiraz in Iran. PPV-specific amplification by polymerase chain reaction (PCR) further confirmed that the disease was associated with PPV infection. Phylogenetic analysis of ORF011 (B2L) gene sequences showed 99.79% and 82.13% similarity of the PPV identified in this study with the Jodhpur isolate and the bovine papular stomatitis virus (BPSV) isolates (CE41), respectively. Moreover, phylogenetic analysis of the ORF045 gene indicated that the Shiraz sample was in all probability closely related to VR634 and to F00.120R and PCPV776. In conclusion, the results suggest that camel PPV (CPPV) is a likely cause of contagious ecthyma in dromedary camels in Iran.

## Introduction

Camel contagious ecthyma (CCE), also known as orf, Auzdik or contagious pustular dermatitis, is a worldwide skin disease in camelids characterised by self-limiting proliferative lesions (Nagarajan et al. 2010). These nodular lesions followed by papules and vesicles mostly appear around the mouth, nares, lips and eyes or in some instances, other sites (Abubakr et al. 2007; Venkatesan et al. 2010). They then develop into scabs and healing may take up to 20 days – 30 days or even more (Nagarajan et al. 2010). This disease is caused by *pseudocowpox virus* (PCPV) in cattle, whereas its main causative agent in sheep, goats, camels and humans is Orf virus (ORFV) (Davari et al. 2013; Nagarajan et al. 2010). However, PCPV has been reported as the etiologic agent of the disease in camels (Abubakr et al. 2007; Nagarajan et al. 2010). The ORFV or CCE virus belongs to the genus *parapoxvirus* (PPV), the subfamily Chordopoxvirinae of the Poxviridae family (Fleming, Wise & Mercer 2015; Harvey et al. 2015; Klein & Tryland 2005; Venkatesan et al. 2010). Morbidity is high, whereas its mortality and fatality rates are low to moderate. Nevertheless, this debilitating disease can lead to high mortality in younger animals due to secondary infections and trouble in suckling and eating (Hosamani et al. 2006). The genome of ORFV contains a linear double-stranded DNA molecule of approximately 134 kbp – 139 kbp in length (Hautaniemi et al. 2010; Li et al. 2013; Rovozze & Burke 1973). The genes at the terminal ends are mostly responsible for viral replication, whereas those located in the central sites are essential for virus virulence and pathogenesis (Li et al. 2013). For instance, the well-conserved ORF045 gene encodes the late transcription factor VLTF-1, whereas the envelope or ORF011 (B2L) gene encodes a highly immunogenic envelope protein stimulating a strong antibody response (Kottaridi et al. 2006; Zhang et al. 2014). ORFV is sometimes transmitted to humans via direct contact with the infected animal (Torfason & Gunadottir 2002). Clinical signs of pox, contagious ecthyma and papillomatosis are similar and difficult to distinguish (Khalafalla, Al-Busada & El-Sabagh 2015a; Khalafalla et al. 2015b; Mosadeghhesari et al. 2014; Nagarajan et al. 2010). Polymerase chain reaction (PCR) and sequencing methods may be useful for genetic characterisation and classification of PPVs (Inoshima et al. 2001; Khalafalla et al. 2015a). The PPVs in Iranian dromedaries (*Camelus dromedarius*) have not yet been characterised at the genetic level, whereas their relationship to PPVs in domestic animals also remains unclear. Also, the ORF011 and ORF045 genes of camel PPV (CPPV) were used for viral detection, examining the outbreak of CPPV infection in camels by PCR and for phylogenetic analysis.

## Material and methods

### Sampling

Sixty skin samples were collected from two outbreaks of ORFV which occurred in 2013 in dromedary camels of Shiraz and Kerman cities, southern and eastern Iran, respectively. Biopsies of the skin and the lip of the affected camels were sent to Razi Research Vaccine and Serum Institute. The samples collected from the diseased camels were analysed for PCR assay.

### DNA extraction and primer design

DNA of the samples from the skin and lip lesions was extracted by High Pure^®^ Extraction Kit (Roche Diagnostics GmbH, Mannheim, Germany). Briefly, 50 mg of the tissue samples was homogenised in 200 μL of phosphate buffer saline (PBS) and mixed with 200 μL of binding buffer and 50 μL of proteinase K then incubated at 72 °C for 10 min. The process was continued according to the manufacturer’s instructions.

According to Inoshima, Morooka and Sentsui (2000), a set of primers including pan-parapoxvirus primer 1 (PPP-1) and PPP-4 were applied based on the sequence of the ORF011 gene. The sequences of PPP-1 and PPP-4 were 5’-gtc gtc cac gat gag cag ct-3’ and 5’-tac gtg gga agc gcc tcg ct-3’, respectively (Inoshima et al. 2000). In addition, the primers 045F (5’-cct act tct cgg agt tca gc-3’) and 045R (5’-gca gca ctt ctc ctc gta g-3’) were applied based on the sequence of 045 gene of ORFV, isolate OV-SA00 (accession no. AY186732) (Delhon et al. 2004).

### Polymerase chain reaction

For amplification of the ORF011 gene, the PCR assay was carried out as follows: 1 μg of DNA isolated from the infected or uninfected cells was added to 50 μL of the PCR mixture containing 0.2 mM primers (PPP-1 and PPP-4), 0.2 mM dATP, dCTP, dGTP and dTTP, 10 mM Tris–HCl (pH 8.3), 50 mM KCl, 1.5 mM MgCl_2_ and 1 U of AmpliTaq Gold DNA polymerase. DNA was amplified with a DNA thermal cycler by a two-step reaction. The mixture was denatured at 95 °C for 9 min, five cycles of 94 °C for 1 min, 50 °C for 1 min, 72 °C for 1 min, 25 repeated cycles at 94 °C for 1 min, 55 °C for 1 min and finally 72 °C for 1 min (Inoshima et al. 2000).

For amplification of the ORF045 gene, the PCR assay was carried out in a reaction mixture of 50 μL containing 1 μg (10 μL) of extracted DNA, 5 μL of dNTP (10 mM), 5 μL of 10x PCR buffer, 2 μL of MgCl_2_ (50 mM), 2 U/tube of Taq polymerase, 50 pmol of each of the primers and 33 μL of distilled water. Forty cycles of denaturation (at 95 °C, for 10 s), annealing (at 47 °C for 10 s) and extension (at 74 °C for 10 s) were followed by a 15-min incubation at 78 °C to final extension of the primers. The amplification products (10 μL) were analysed by 2% agarose gel in Tris–boric acid–EDTA (TBE) buffer and stained with SYBR Green (1 μg/mL) (Kottaridi et al. 2006). The amplicons were visualised using a UV transilluminator ‘gel doc’ system.

### Sequencing of the polymerase chain reaction products containing ORF011 and ORF045 genes

The PCR products with the expected sizes (594 bp and 393 bp) were purified from the gel using the Bioneer (Daejeon, South Korea) gel extraction kit, according to the manufacturer’s procedures, and then were sequenced by the use of neighbour-joining method (Bioneer).

### Phylogenetic analysis

Comparison among the isolates of this study and those of the ORFV available in GenBank, NCBI database, was made using the online BLAST program. Sequence identities of the nucleotides were analysed by CLC program version 5.5. The sequences were assembled into multiple sequence alignment. A phylogenetic tree derived from the nucleotide sequences was constructed for the ORFV, using neighbour-joining method of CLC program version 5.5. The sequences of the ORF011 and ORF045 genes of ORFV used in phylogenetic analysis are presented in Tables 1 and 2.

**TABLE 1:** Nucleotide sequences of ORF011 gene that were aligned in the samples of the present study.

Serial number	Virus isolate (strain)	Organism	Country	Reference	Accession number
1	SV252/11	ORFV	Brazil	Schmidt et al. (2013)	JX485997.1
2	SV178/12	ORFV	Brazil	Schmidt et al. (2013)	JX485994.1
3	MT-05	ORFV	Brazil	DS	JN613809.1
4	MT-05	ORFV	Brazil	Abrahao et al. (2009)	FJ665818.1
5	Ovino_Patos	ORFV	Brazil	Schmidt et al. (2013)	JX485993.1
6	Cangucu	ORFV	Brazil	Schmidt et al. (2013)	JX485989.1
7	Jodhpur	Camel contagious ecthyma virus	India	Lucinda et al. (2010)	GQ390365.1
8	CE41	Bovine papular stomatitis virus	Sudan	Dal Pozzo et al. (2011)	JN171861.1
9	PA11	ORFV	Brazil	De Oliveira et al. (2012)	JQ349520.1
10	SV29/12	ORFV	Brazil	Schmidt et al. (2013)	JX485991.1
11	SV26/12	ORFV	Brazil	Schmidt et al. (2013)	JX485988.1
12	SV561/11	ORFV	Brazil	Schmidt et al. (2013)	JX485990.1
13	SV27/12	ORFV	Brazil	Schmidt et al. (2013)	JX485995.1
14	SV28/12	ORFV	Brazil	Schmidt et al. (2013)	JX485996.1
15	SV520/11	ORFV	Brazil	Schmidt et al. (2013)	JX485986.1
16	PCPV776	Pseudocowpox virus	Austria	DS	HM589036.1
17	48/01	Pseudocowpox virus	Austria	DS	AY636048.1
18	Cam/09	ORFV	India	DS	GU460370.1
19	NE1	ORFV	Brazil	Abrahao et al. (2009)	FJ665819.1
20	NE1	ORFV	Brazil	DS	JN613810.1
21	Bahia	ORFV	Brazil	Schmidt et al. (2013)	JX485987.1
22	Caprino_SJE	ORFV	Brazil	Schmidt et al. (2013)	JX485992.1
23	Taiping	ORFV	Taiwan	Chan et al. (2009)	EU327506.1

ORFV, Orf virus; DS, direct submission.

**TABLE 2:** Nucleotide sequences of ORF045 gene that were aligned in the samples of the present study.

Serial number	Virus isolate (strain)	Organism	Country	Reference	Accession number
1	BV- AR02/ORFB	Bovine papular stomatitis virus	United States: Arkansas	Delhon et al. (2004)	AY386265.1
2	VR634	Pseudocowpox virus	Finland	Hautaniemi et al. (2010)	GQ329670.1
3	F00.120R	Pseudocowpox virus	Finland	Hautaniemi et al. (2010)	GQ329669.1
4	PCPV776	Pseudocowpox virus	Austria	DS	HM589037.1
5	Shiraz1	ORFV	Iran	-	KC534486.1
6	Shiraz3	ORFV	Iran	-	KC534488.1
7	OV-SA00/ORFD	ORFV	United States: Texas	Delhon et al. (2004)	AY386264.1
8	Shiraz2	ORFV	Iran	-	KC534487.1
9	Egypt2006	ORFV	Egypt	Mahmoud, Abdelrahman and Soliman (2010)	EU826136.1
10	Shiraz5	ORFV	Iran	-	KC534490.1
11	OV-IA82/ORFA	ORFV	United States: Iowa	Delhon et al. (2004)	AY386263.1
12	NZ2	ORFV	New Zealand	Mercer et al. (2006)	DQ184476.1
13	D1701	ORFV	Germany	McGuire, Johnston and Sykes (2012)	HM133903.1
14	Shiraz4	ORFV	Iran	-	KC534489.1

ORFV, Orf virus; DS, direct submission.

## Results

### Polymerase chain reaction and DNA sequencing

The PCR method was performed to amplify the ORF011 gene. This method successfully detected the DNA from members of the PPV genus and was able to diagnose the ORFV infection. To confirm the causative agent, a partial region of the ORF011 gene was amplified. The primers PPP-1 and PPP-4 were described as the universal primers to amplify most species of ORFVs that have been used in the present study. A 594-bp product representing the region (157 nt – 750 nt) of the ORF011 gene was amplified from Shiraz samples. The 594-bp DNA fragment was sequenced and the nucleotide sequence data confirmed ORFV. Another PCR test was performed on DNA purified from Kerman samples as template and the pair of 045F/045R primers to amplify the ORF045 gene. PCR resulted in the amplification of the gene ORF045 with an expected DNA fragment (392 bp).

### Phylogenetic analysis

**FIGURE 1:**
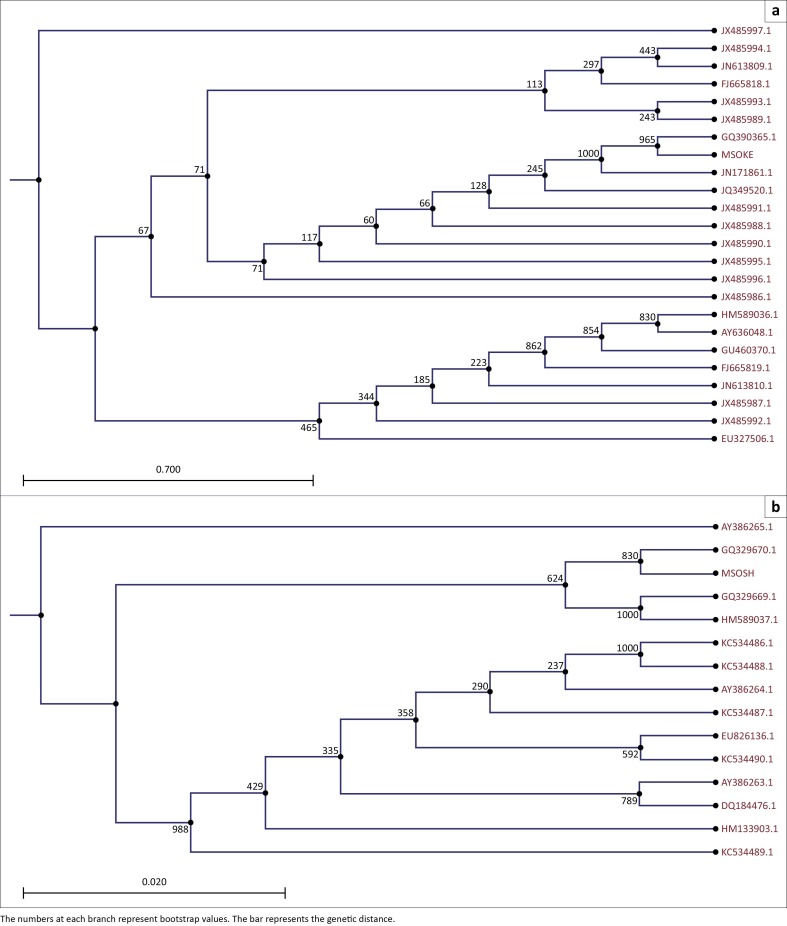
Phylogenetic analysis of the Orf virus based on the (a) ORF011 and the (b) ORF045 gene. The phylogenetic trees were constructed using the neighbour-joining algorithm in CLC Main workbench 5.5 with 1000 bootstrap replicates.

The samples used in this study included Kerman (MSOKE) and Shiraz (MSOSH) isolates. The sequences aligned with MSOKE and MSOSH were based on the ORF011 gene (Table 1) and the gene ORF045 (Table 2), respectively. As stated previously, the samples of this study and the previous isolates were aligned and the phylogenetic tree was drawn by CLC program version 5.5. Results of the phylogenetic tree related to the ORF011 gene have been shown in Figure 1a that there is a main clade or lineage containing SV252/11 and other isolates. A branch of the main clade contains three clades including MSOKE and CCE virus isolate from Jodhpur, MSOKE/Jodhpur isolate sister to bovine papular stomatitis virus (BPSV) isolate CE41, and Clade MSOKE/Jodhpur and CE41 sisters to Brazil isolates. MSOKE had 99.77% and 82.13% similarity with Jodhpur and CE41 isolates, respectively. In addition, MSOKE had minimum distance (0.20) to Jodhpur and CE41. The results of phylogenetic tree from gene ORF045 with two principal clades analysis are shown in Figure 1b including clade ORFB and other sequences, clade GQ329670/MSOSH and GQ329669/HM589037 while another branch contains other sequences. Indeed, MSOSH was closely related to PCPV from Finland (VR634), another PCPV from Finland (F00.120R) and then also to PCPV from Austria *(*PCPV776).

## Discussion

Several procedures have been developed to detect ORFV, while most of them are time-consuming, costly and sometimes associated with low specificity, reduced sensitivity and with cross reactions (Inoshima et al. 2001; Khalafalla et al. 2015a; Kottaridi et al. 2006). Assays based on PCR are considered as rapid, sensitive and specific tests for identifying the causative agents of diseases (Andrade et al. 1983; Inoshima et al. 2001, 2002). Several PCR protocols have been described for detecting the DNA of PPV (Inoshima et al. 2000, 2001, 2002; Torfason & Gunadottir 2002). Two target genes to generate PCR amplicons from CCE DNA for sequence analysis are the open reading frame ORF011 and ORF045 which encode VLTF-1 (Khalafalla et al. 2015b; Kottaridi et al. 2006). Sequencing of the suspected samples showed that MSOSH had close relation to PCPV from Finland (VR634) followed by another PCPV from Finland (F00.120R) and PCPV from Austria (PCPV776). Additionally, MSOKE had 99.77% and 82.13% similarity to Jodhpur and CE41 isolates, respectively. Khalafalla et al. (2015b) considered that camel ORFV isolates can be divided into two genetic clades including the Asian lineage of isolates from Saudi Arabia, Bahrain and India and the African lineage of isolates from Sudan, based on the sequence of the ORF011 gene among camels in Sudan. They showed that the camel ORFV is diverse from viruses close to PCPV and ORFV. Nagarajan et al. (2010) analysed and compared the sequence of Indian PCPV with several sequences related to ORFV, PCPV and BPSV available in the database. They showed that ORFV from different regions of the world had 92.3% – 92.7% and 96.2% – 96.5% sequence similarity with camel PCPV at the nucleotide and amino acid level, respectively.

ORFV has previously been cultivated on cell cultures such as Vero, lamb testis (LT) and ovine testis (OA3.Ts), whereas the cell culture was done on chicken embryo fibroblast (CEF) in this study. It is because CPE includes cell detachment, rounded cells and aggregation of cells. Meanwhile, many researchers have studied the effects of ORFV on various cell cultures. Housawi et al. (2012) compared the immunogenicity and protective efficacy of three locally developed live ORFV vaccines in Vero cell culture. For production of a live attenuated vaccine against ORFV, a wild virus strain has been attenuated through serial passages on the primary CEF tissue cultures (Mercante et al. 2008). The isolation and attenuation of the virus in Iran would therefore be necessary for protection of camels. A recent study performed by Mombeni et al. (2013) in Iran reported severe cases of CCE that caused a morbidity and mortality rate of 70.6% and 6.0%, respectively. This points to the potential importance of this disease in Iran. This study has indicated that ORFV with an apparent close similarity to PCPV can be a major cause of contagious ecthyma in Iranian camels.
